# Cannabis Use and Its Interaction with Anxiety Disorders

**DOI:** 10.1192/j.eurpsy.2024.876

**Published:** 2024-08-27

**Authors:** F. Varino, T. Duarte, F. S. Silva, P. Froes, A. M. Fernandes, P. Baronet

**Affiliations:** Psychiatry, Centro Hospitalar Universitário Lisboa Norte, Lisbon, Portugal

## Abstract

**Introduction:**

Cannabis use has been reported to cause a myriad of acute adverse reactions, including those linked to anxiety disorders, such as panic attacks and derealization. Notably, in the emergency department, anxiety makes up a significant proportion of the complaints related to cannabinoid consumption. Several reports show these symptoms can persist after the cessation of cannabis consumption. Consequently, some questions have arisen regarding the role of cannabinoids as precipitators for anxiety disorders in vulnerable individuals. Alternatively, it has been hypothesized that patients with anxiety disorders are more prone to using cannabis.

**Objectives:**

We aim to understand whether there is an established relationship between anxiety disorders and cannabis use. Moreover, we intend to identify what are the factors which make an individual more likely to experience anxiety following cannabis consumption.

**Methods:**

A search was conducted in the PubMed database using the MeSH terms “cannabis”, “panic disorder”, “anxiety”, “panic” and “generalized anxiety disorder”. Articles published in the last ten years were considered. Publications were selected after careful reading of their abstract. A non-systematic review of the selected articles was performed.

**Results:**

Eight articles were included in this review. While a majority of these publications did not find a significant association between cannabis use and anxiety disorders, a small subset of analyzed articles found that cannabis use may increase anxiety severity in general, devoid of specific diagnostic association. Individuals who presented to the emergency department with anxiety complaints after cannabis use were likely to be young and to have ingested edible cannabis. History of psychiatric disease, especially substance use disorder, was common in this population.

**Conclusions:**

Most available data suggest cannabis use is not clearly linked to anxiety disorders. However, information around this topic is scarce and heterogenous. Further research is needed focusing on the natural evolution of acute anxiety after cannabis use. Factors such as young age, presence of psychiatric comorbidities and consumption of edible cannabis appear to contribute to a significantly increased risk of experiencing acute anxiety after cannabis use.

**Disclosure of Interest:**

None Declared

